# Application study of an artificial intelligence and big data-based personalized chronic disease management model for diabetes patients

**DOI:** 10.3389/fpubh.2026.1735295

**Published:** 2026-01-29

**Authors:** Mei Xin, Yanbing Yao, Ping Huang, Qiuxia Li

**Affiliations:** 1 Department of Health Management, The First Affiliated Hospital of Guangxi Medical University, Nanning, Guangxi, China; 2 Department of Elderly Endocrinology, The First Affiliated Hospital of Guangxi Medical University, Nanning, Guangxi, China

**Keywords:** artificial intelligence, big data analytics, glycemic control, personalized diabetes management, quality of life, self-care activities

## Abstract

**Background:**

To evaluate the real-world effectiveness of an artificial intelligence (AI) and big data-driven personalized chronic disease management model for type 2 diabetes mellitus (T2DM) patients, compared to conventional nurse-led management, and to identify factors associated with successful glycemic control within the personalized model.

**Methods:**

A retrospective cohort study was conducted involving 280 T2DM patients discharged from a single hospital between January 2019 and December 2024. Patients were divided into a conventional management group (*n* = 100) and a personalized management group (*n* = 180). The personalized group utilized a model integrating gradient boosting (XGBoost) for risk prediction and rule-based reasoning with reinforcement learning to dynamically generate individualized dietary, exercise, and blood glucose monitoring plans via a mobile application (APP). Both groups received 6 months of follow-up. Glycemic control [fasting blood glucose (FBG), 2-h postprandial glucose (2hPG), glycated hemoglobin (HbA1c)], self-care activities [Summary of Diabetes Self-Care Activities (SDSCA) scale], and quality of life [Diabetes-Specific Quality of Life (DSQL) scale] were assessed at baseline and 6 months. Within the personalized group, patients were further categorized into well-controlled (HbA1c ≤ 6.5%, *n* = 98) and poorly-controlled (HbA1c > 6.5%, *n* = 82) subgroups for case–control analysis.

**Results:**

At 6 months, the personalized management group demonstrated significantly better glycemic control (FBG: 6.79 ± 0.72 vs. 7.03 ± 0.89 mmol/L, *p* = 0.022; 2hPG: 6.27 ± 1.18 vs. 6.62 ± 1.16 mmol/L, *p* = 0.018; HbA1c: 6.48 ± 0.53% vs. 6.63 ± 0.46%, *p* = 0.018), superior self-care scores across all SDSCA domains (all *p* < 0.05, largest improvement in special diet: *p* = 0.001), and significantly higher quality of life (all DSQL dimensions *p* < 0.05) compared to the conventional group. Within the personalized group, multivariate analysis identified alcohol consumption [odds ratio (OR) = 3.576, *p* < 0.001], low baseline high-density lipoprotein cholesterol (HDL-C) (OR = 0.102, *p* = 0.007), and reduced blood glucose monitoring adherence (OR = 0.958, *p* < 0.001) as independent risk factors for poor control, while higher exercise plan completion was protective (OR = 0.976, *p* = 0.037).

**Conclusion:**

The AI and big data-driven personalized management model significantly improved glycemic control, self-care behaviors, and quality of life in T2DM patients over conventional care within 6 months. Success within the model is influenced by behavioral and biological factors, alongside alcohol consumption. This approach demonstrates promise for enhancing diabetes care.

## 1 Introduction

Diabetes mellitus, a chronic metabolic disorder marked by hyperglycemia due to defects in insulin secretion or action, poses a significant global health challenge. With an estimated 537 million adults affected in 2021, this number is projected to rise to 783 million by 2045 (1–3). The condition’s long-term complications, such as retinopathy, nephropathy, neuropathy, cardiovascular disease, and stroke, substantially burden individuals and healthcare systems, impacting morbidity, mortality, and quality of life while generating immense economic costs (1, 4). Effective long-term management is crucial for glycemic control and for preventing or delaying these severe outcomes.

Achieving optimal glycemic targets extends beyond pharmacotherapy, requiring sustained patient engagement in multifaceted self-care. This includes individualized dietary management, consistent physical activity, regular blood glucose monitoring, medication adherence, foot care, and adaptive problem-solving (5). However, maintaining these behaviors long-term proves challenging due to regimen complexity, knowledge gaps, psychological barriers, socioeconomic constraints, and inadequate support (6, 7). Consequently, suboptimal glycemic control remains prevalent, driving adverse outcomes (8, 9).

Conventional diabetes management, often reliant on periodic clinic visits and standardized education, struggles to address individual variability. These models provide generalized advice but lack the timeliness and personalization needed for sustained behavioral adaptation (10). The “one-size-fits-all” approach fails to account for differences in pathophysiology, lifestyle, comorbidities, psychosocial context, and treatment response (11). Furthermore, episodic care limits real-time data capture and proactive intervention between visits, exacerbating disparities in low-resource settings (12).

The emergence of artificial intelligence (AI) and big data analytics offers unprecedented potential to overcome these limitations. AI algorithms can analyze multi-dimensional datasets—including electronic health records, continuous glucose monitoring, wearable device data, and socio-behavioral information—to uncover intricate patterns and individual risk profiles (13, 14). This enables personalized chronic disease management through real-time risk prediction, tailored self-management recommendations (e.g., precision nutrition/exercise plans), and adaptive feedback (15). Big data infrastructure supports the aggregation and secure processing of heterogeneous data, forming the foundation for proactive interventions and resource optimization (16).

While the theoretical promise of AI and big data in personalized diabetes management is immense, rigorous evaluation of its real-world implementation and effectiveness within established healthcare settings is crucial before widespread adoption can be justified. Translating algorithmic potential into tangible improvements in patient outcomes requires robust application studies that assess not only clinical endpoints like glycemic control but also critical factors such as patient self-care behaviors, quality of life, feasibility, and the identification of factors influencing success within personalized models. Understanding how these advanced tools integrate with and enhance existing clinical workflows, particularly nursing-led management, is essential.

This study aims to address this critical gap by conducting an application study of an AI and big data-based personalized chronic disease management model specifically designed for diabetes patients. Furthermore, the study seeks to identify factors associated with successful glycemic control within the personalized management framework itself. The ultimate goal is to provide empirical evidence on the practical value and potential benefits of integrating advanced AI-driven personalization into the ongoing care of individuals living with diabetes.

## 2 Materials and methods

### 2.1 Study design and selection criteria

A retrospective analysis was conducted on 280 diabetes patients admitted to our hospital from January 2019 to December 2024. Based on different post-discharge management strategies, the diabetes patients (*n* = 280) were divided into a conventional management group (*n* = 100) and a personalized management group (*n* = 180). All patients were managed and followed up for 6 months. According to the blood glucose control status 6 months after discharge, patients in the personalized management group (*n* = 180) were further categorized into a well-controlled group (*n* = 98) and a poorly controlled group (*n* = 82). The well-controlled group was defined as having a glycated hemoglobin (HbA1c) level ≤6.5%, while the poorly controlled group was defined as having an HbA1c level >6.5% (17).

Inclusion criteria included: ① Meeting the diagnostic criteria for Type 2 Diabetes Mellitus (T2DM) (18); ② Age ≥18 years; ③ Duration of diabetes <10 years; ④ Ability to perform daily activities independently and use a smartphone to access the internet; ⑤ Complete electronic health records and questionnaires without missing data. Exclusion criteria included: ① Pregnant or lactating women; ② Individuals with mental or psychological disorders; ③ Presence of malignant tumors or severe complications.

### 2.2 Conventional management strategy led by nurses in the physical examination department

#### 2.2.1 Health education and material distribution

Within 48 h of patient admission, specialized nurses from the physical examination department conducted group lectures (8–10 patients per group) combined with one-on-one explanations to provide education on basic diabetes knowledge, dietary principles (daily calorie control and food pairing ratios), exercise safety (appropriate exercise intensity and contraindications), and blood glucose monitoring methods (proper fingerstick blood collection and glucometer operation). Simultaneously, the “Diabetes Self-Management Handbook” (including sample meal plans, exercise logs, and blood glucose monitoring log templates) was distributed, following guidelines provided by the American Diabetes Association (2021) (19).

#### 2.2.2 Follow-up

During the first 3 months after discharge, health reminders (dietary precautions and exercise recommendations) were sent once a week via the hospital’s chronic disease management platform. Two telephone follow-ups were conducted each month, covering topics such as adherence to hypoglycemic medications, dietary compliance, exercise frequency and duration, and blood glucose monitoring data. Patient-reported discomfort symptoms were also recorded. From the 4th to the 6th month after discharge, monthly telephone follow-ups were conducted with the same content. Every 3 months, an outpatient follow-up appointment was scheduled, during which fasting blood glucose and glycated hemoglobin tests were performed with the assistance of the physical examination department nurses, and paper copies of the blood glucose monitoring logs were collected.

### 2.3 Personalized chronic disease management model

First, based on multi-source big data, 15 core features were extracted as input variables for the model. These 15 core features are Age, Gender, Body Mass Index (BMI), Baseline glycated hemoglobin (HbA1c), Baseline fasting blood glucose (FBG), Duration of diabetes, Smoking status, Alcohol consumption history, High-density lipoprotein cholesterol (HDL-C), Triglyceride (TG) level, Physical activity level, Diet compliance, Frequency of blood glucose monitoring, Medication adherence, Presence of comorbidities.

To develop the risk prediction model, we evaluated the performance of several machine learning algorithms using the same training dataset and input features. The algorithms tested included logistic regression, support vector machine (SVM), random forest, and XGBoost (gradient boosting).

Model performance was assessed using 5-fold cross-validation. Evaluation metrics included accuracy, precision, recall, F1-score, and area under the receiver operating characteristic curve (AUC). Among the tested models, XGBoost demonstrated the highest AUC (0.861), the best balance between precision and recall, and superior performance in detecting high-risk patients with poor glycemic control. As a result, the XGBoost model was selected for the risk prediction submodule. Then, using the Python 3.8 platform and the Scikit-learn library, multiple algorithm performances were compared to select the optimal model:

#### 2.3.1 Risk prediction submodel

A gradient boosting tree (XGBoost) algorithm was used to construct a model for predicting the risk of poor blood glucose control. The dependent variable was “whether HbA1c levels were within target range within 6 months after discharge,” with the aforementioned 15 features as inputs. Hyperparameters were optimized using 5-fold cross-validation (learning rate of 0.1 and tree depth of 5), which ultimately enabled the accurate identification of high-risk patients with poor blood glucose control.

#### 2.3.2 Personalized management plan generation submodel

Combining rule-based reasoning and reinforcement learning (RL) algorithms, this submodel uses risk prediction results as a foundation to integrate conventional management plans (Section 2.2) and construct a knowledge graph. It dynamically generates intervention plans for high-risk patients:

1. Dietary plan: Based on the patient’s BMI, daily exercise volume, and dietary preferences (analyzed through the APP’s dietary record), the model calculates the daily caloric needs (e.g., 25–30 kcal/kg of ideal body weight) and allocates macronutrient proportions (carbohydrates: 50–60%, proteins: 15–20%, fats: 20–30%). Specific meal plans (e.g., breakfast: whole wheat bread + eggs; lunch: mixed grain rice + lean meat + leafy greens) are generated and pushed to the patient via the mobile APP.
2. Exercise plan: Considering the patient’s age, comorbidities (e.g., avoiding intense exercise for patients with hypertension), and exercise habits, the model recommends moderate-intensity exercise types (e.g., brisk walking, Tai Chi) and durations (e.g., starting with 20 min per session, five times a week, gradually increasing to 30 min). The phone’s alarm clock is set to remind patients of their exercise schedule.
3. Blood glucose monitoring plan: Based on blood glucose fluctuation levels (e.g., recent fasting blood glucose fluctuations >2 mmol/L), the model adjusts the monitoring frequency (e.g., adding one bedtime measurement). The APP automatically generates a monitoring schedule.

### 2.4 Effectiveness evaluation

#### 2.4.1 Electronic medical records

Accessing the hospital’s electronic medical record system (HIS), we retrieved and extracted information on diabetes patients at the time of admission, including age, gender, body mass index (BMI), education level, payment method for medical expenses, underlying diseases, smoking history, alcohol consumption history, marital status, and waist circumference.

#### 2.4.2 Laboratory testing data

At the time of admission, an automatic biochemical analyzer (AU480, Beckman Coulter, United States) was used to measure levels of triglycerides (TG), high-density lipoprotein (HDL), total cholesterol (TC), low-density lipoprotein (LDL), serum creatinine (Scr), urea (BUN), total bilirubin (TBIL), alanine aminotransferase (ALT), and aspartate aminotransferase (AST).

#### 2.4.3 Summary of diabetes self-care activities

Six months after discharge, patients of diabetes filled in the SDSCA online through the hospital’s official account to assess self-management behavior. The SDSCA includes six dimensions: general diet, special diet, exercise, blood glucose monitoring, foot care, and medication, with a total of 11 items. Each item is scored from 0 to 7, with higher scores indicating better self-management. The Cronbach’s *α* coefficient for this scale is 0.63 (20).

#### 2.4.4 Diabetes-specific quality of life scale

The DSQL was used to assess the quality of life of diabetes patients at the 3-month and 6-month follow-up visits after discharge. The DSQL covers four dimensions: psychological function, physiological function, treatment effectiveness, and social relationships, with a total of 27 items. Each item is rated on a 5-point Likert scale ranging from “completely disagree” (1 point) to “completely agree” (5 points). Lower scores indicate a higher quality of life. The Cronbach’s *α* coefficient for this scale is 0.91 (21).

### 2.5 Moral statement

This study strictly adhered to the Declaration of Helsinki and the ethical guidelines for medical research. All procedures were approved by the hospital’s medical ethics committee. All patient data, including electronic medical records, laboratory test results, dynamic data collected via smartphone, and scale scores, were anonymized using a unique patient identifier (ID). The raw data were encrypted and stored on a dedicated hospital server, accessible only to authorized research team members (such as nurses from the physical examination department and statistical analysts) after permission verification.

### 2.6 Statistical analysis

All data in this study were analyzed using SPSS statistical software (version 29.0; developed by SPSS Inc., Chicago, IL, United States). Continuous variables that passed normality tests were expressed as mean ± standard deviation (M ± SD), and group comparisons were performed using independent samples t-tests. Categorical variables were expressed as percentages (%), and group comparisons were conducted using χ^2^ tests. A *p* < 0.05 was considered statistically significant. Variables with a *p* < 0.05 difference between the case–control group under personalized management were used as independent variables, with poor blood glucose control as the dependent variable, to perform multivariate logistic regression analysis to identify independent risk factors for poor blood glucose control under personalized management.

## 3 Results

### 3.1 Cohort study

#### 3.1.1 Basic data

Tables 1, 2 demonstrate no statistically significant differences in demographic characteristics (including age, gender, BMI, educational level, payment method for medical expenses, underlying disease, smoking history, drinking history, marital status, waist circumference, course of disease) or laboratory parameters (including TG, TC, LDL, HDL, Scr, BUN, TBIL, ALT, AST) between the conventional management group (*n* = 100) and the personalized management group (*n* = 180) at baseline (all *p* > 0.05). This confirms the comparability of the two cohorts before intervention.

**Table 1:** Comparison of demographic characteristics of the conventional management group and the personalized management group.

Parameter	Conventional management group (*n* = 100)	Personalized management group (*n* = 180)	t/χ^2^	*P*
Age (years)	47.32 ± 7.15	46.87 ± 7.43	0.496	0.620
Gender [n(%)]			0.039	0.843
Male	56 (56.00%)	103 (57.22%)		
Famale	44 (44.00%)	77 (42.78%)		
BMI (kg/m^2^)	27.15 ± 2.84	27.03 ± 3.02	0.326	0.744
Educational level [n(%)]			0.305	0.58
High school and above	46 (46.00%)	89 (49.44%)		
Below high school	54 (54.00%)	91 (50.56%)		
Payment method for medical expenses [n(%)]			0.387	0.534
Urban employee medical insurance	59 (59.00%)	113 (62.78%)		
Urban and rural residents’ medical insurance	41 (41.00%)	67 (37.22%)		
Underlying disease [n(%)]				
Hypertension	56 (56.00%)	105 (58.33%)	0.143	0.705
Hyperlipidemia	49 (49.00%)	91 (50.56%)	0.062	0.803
Smoking history [n(%)]			0.557	0.456
Yes	29 (29.00%)	60 (33.33%)		
No	71 (71.00%)	120 (66.67%)		
Drinking history [n(%)]			0.583	0.445
Yes	37 (37.00%)	75 (41.67%)		
No	63 (63.00%)	105 (58.33%)		
Marital status [n(%)]			1.237	0.539
Married	78 (78.00%)	149 (82.78%)		
Unmarried	7 (7.00%)	12 (6.67%)		
Divorced or widowed	15 (15.00%)	19 (10.56%)		
Waist circumference (cm)	89.27 ± 5.32	88.94 ± 5.18	0.501	0.617
Course of disease (years)	5.12 ± 1.47	5.07 ± 1.52	0.289	0.773

BMI, body mass index.

**Table 2:** Comparison of laboratory features of the conventional management group and the personalized management group.

Parameter	Conventional management group (*n* = 100)	Personalized management group (*n* = 180)	*t*	*P*
TG (mmol/L)	2.85 ± 0.61	2.82 ± 0.84	0.343	0.732
TC (mmol/L)	4.23 ± 1.05	4.17 ± 1.32	0.412	0.681
LDL (mmol/L)	2.58 ± 0.51	2.54 ± 0.73	0.520	0.603
HDL (mmol/L)	1.13 ± 0.23	1.17 ± 0.21	1.487	0.138
Scr (μmol/L)	115.21 ± 14.09	118.76 ± 16.32	1.829	0.069
BUN (mmol/L)	5.85 ± 0.84	6.07 ± 1.02	1.884	0.061
TBIL (μmol/L)	10.66 ± 2.18	11.02 ± 1.24	1.536	0.127
ALT (U/L)	28.35 ± 6.72	29.47 ± 8.93	1.187	0.236
AST (U/L)	25.61 ± 5.24	26.86 ± 7.15	1.680	0.094

TG, triglyceride; TC, total cholesterol; LDL, low density lipoprotein; HDL, high density lipoprotein; Scr, serum creatinine; BUN, blood urea nitrogen; TBIL, total bilirubin; ALT, alanine aminotransferase; AST, aspartate aminotransferase.

#### 3.1.2 Blood glucose control indicators

**Figure 1:**
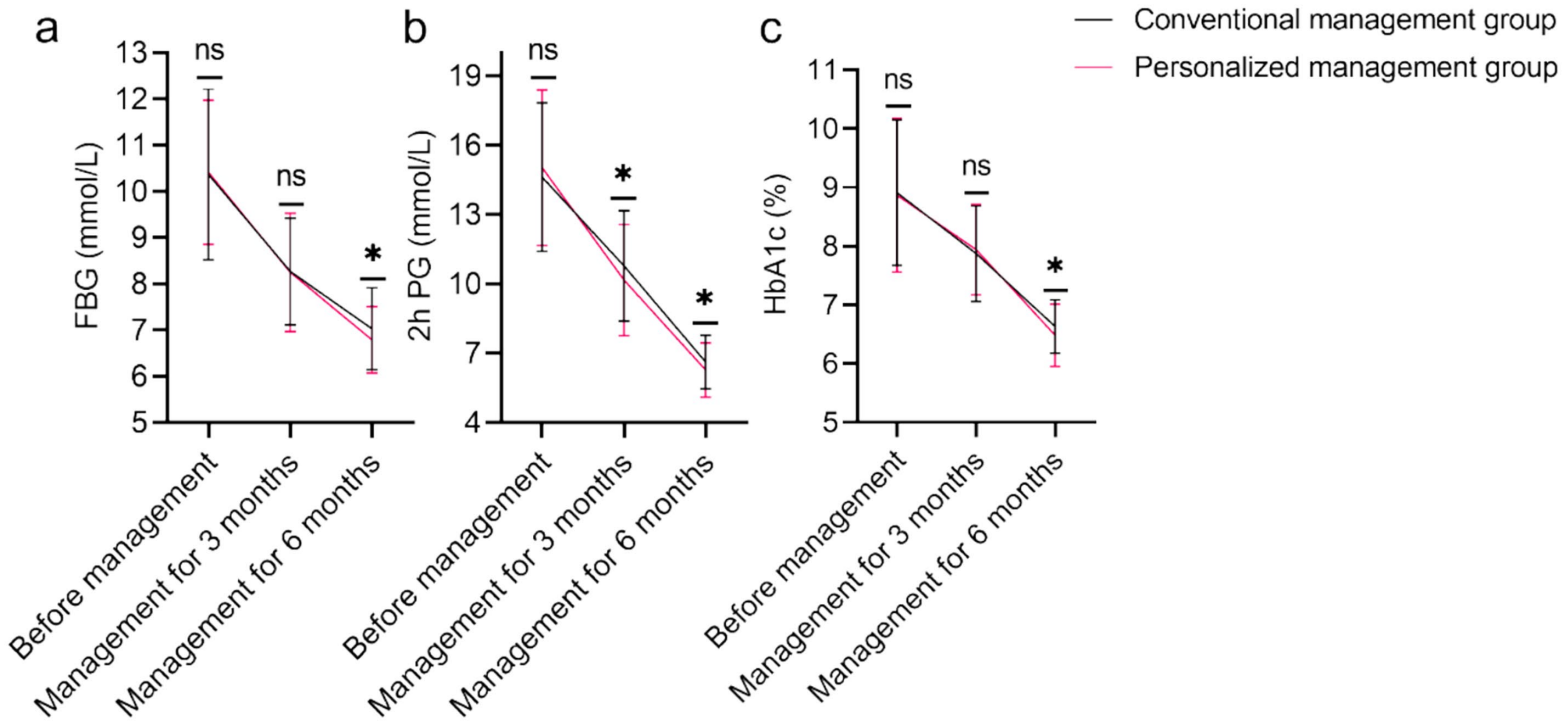
Comparison of blood glucose control indicators of the conventional management group and the personalized management group. **(a)** FBG (mmol/L); **(b)** 2 h PG (mmol/L); **(c)** HbA1c (%). FBG, Fasting blood glucose; 2 h PG, 2-h postprandial glucose; HbA1c, Hemoglobin A1c; ns, No significant difference; *p < 0.05.

Figure 1 indicates no baseline differences in FBG (*p* = 0.808), 2 h PG (*p* = 0.317), or HbA1c (*p* = 0.801). Differences at 3 months were non-significant except for 2 h PG (*p* = 0.041). After 6 months, significantly better glycemic control in the personalized management group after 6 months: lower FBG (7.03 ± 0.89 vs. 6.79 ± 0.72 mmol/L, *p* = 0.022), 2 h PG (6.62 ± 1.16 vs. 6.27 ± 1.18 mmol/L, *p* = 0.018), and HbA1c (6.63 ± 0.46% vs. 6.48 ± 0.53%, *p* = 0.018).

### 3.2 Self-care activities

Table 3 shows significantly higher self-care scores across all domains (general diet, exercise, blood glucose monitoring, foot care, special diet, and medication) in the personalized management group compared to conventional management after 6 months (all *p* < 0.05), with the largest improvement in special diet (*p* = 0.001).

**Table 3:** Comparison of self-care activities of the conventional management group and the personalized management group (scores).

Parameter	Conventional management group (*n* = 100)	Personalized management group (*n* = 180)	*t*	*P*
General diet	9.69 ± 1.87	10.26 ± 1.53	2.592	0.010
Exercise	9.08 ± 2.03	9.63 ± 1.85	2.336	0.020
Blood glucose monitoring	7.29 ± 1.73	7.84 ± 1.32	2.725	0.007
Foot care	8.26 ± 1.62	8.75 ± 1.38	2.688	0.008
Special diet	10.16 ± 2.04	10.98 ± 2.03	3.212	0.001
Medication	6.62 ± 0.68	6.89 ± 0.75	3.013	0.003

Overall adherence to self-management practices, as measured by the SDSCA, was significantly higher in the personalized management group across all domains, with particularly notable improvements in dietary and blood glucose monitoring behaviors. In addition, within the personalized group, adherence indicators derived from APP usage, such as login frequency, dietary/exercise plan completion, and blood glucose monitoring rates, were significantly associated with glycemic control outcomes.

### 3.3 Quality of life

**Figure 2:**
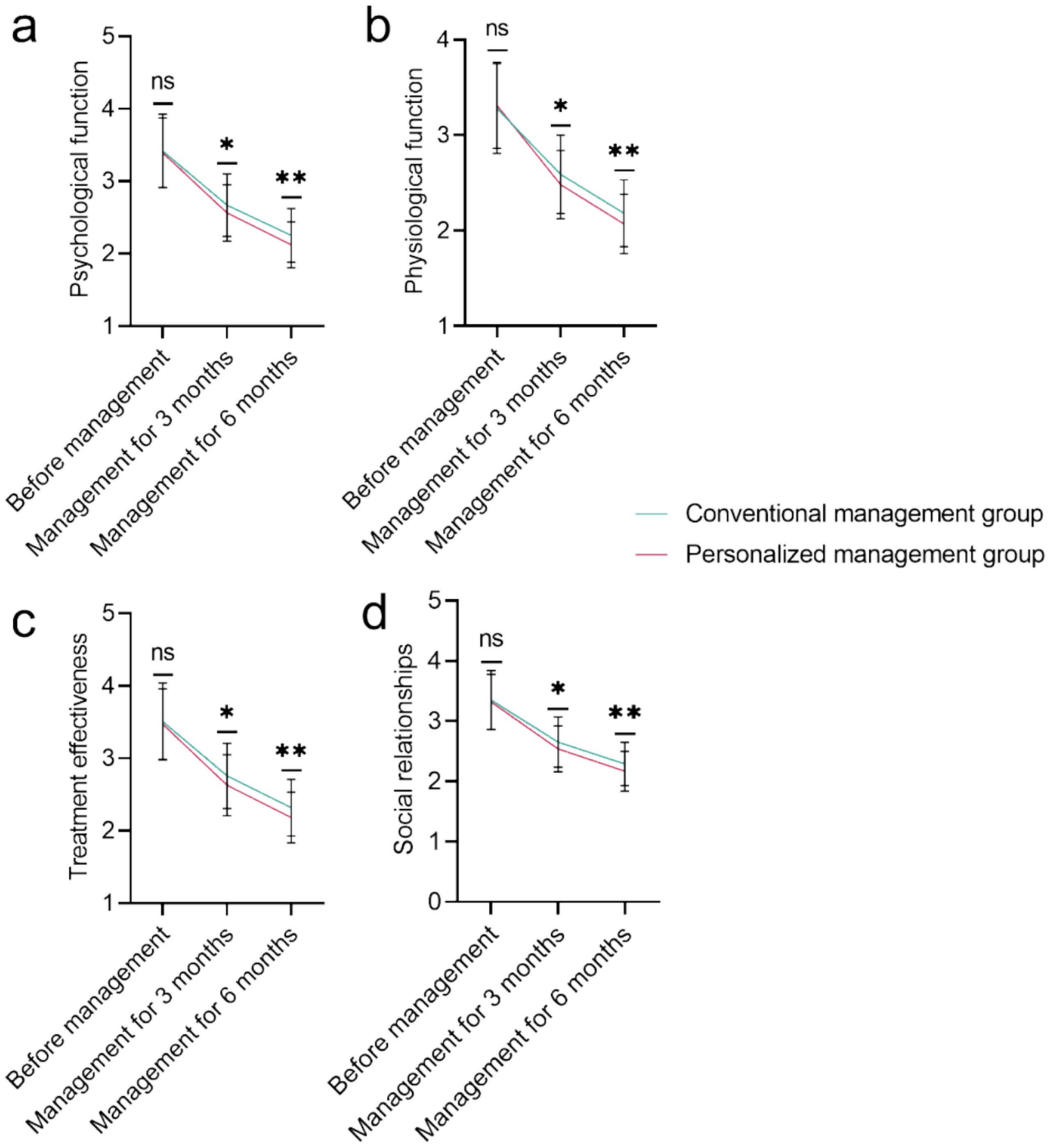
Comparison of the quality of life of the conventional management group and the personalized management group (scores): **(A)** Psychological function; **(B)** Physiological function; **(C)** Treatment effectiveness; **(D)** Social relationships. NS, No significant difference; *p < 0.05; **p < 0.01.

Figure 2 demonstrates no baseline differences in DSQL dimensions (psychological: *p* = 0.625; physiological: *p* = 0.608; treatment: *p* = 0.517; social: *p* = 0.638). Superior quality of life in the personalized group at 3 and 6 months across all DSQL dimensions (psychological, physiological, treatment effectiveness, social relationships; all *p* < 0.05). The most pronounced difference was in psychological function at 6 months (2.25 ± 0.37 vs. 2.12 ± 0.32, *p* = 0.002).

### 3.4 Case control study under personalized management

#### 3.4.1 Baseline characteristics

Table 4 identifies lower baseline HDL (1.16 ± 0.21 vs. 1.06 ± 0.23 mmol/L, *p* = 0.002) and higher alcohol consumption (31.63% vs. 53.66%, *p* = 0.003) in the poorly controlled subgroup (*n* = 82) compared to the well-controlled subgroup (*n* = 98).

**Table 4:** Comparison of baseline characteristics of the well-controlled group and the poorly controlled group under personalized management.

Parameter	Well-controlled group (*n* = 98)	Poorly controlled group (*n* = 82)	t/χ^2^	*P*
Age (years)	46.12 ± 7.21	47.89 ± 7.65	1.594	0.113
Male/Famale [n(%)]	53 (54.08%) / 45 (45.92%)	50 (60.98%) / 32 (39.02%)	0.867	0.352
BMI (kg/m^2^)	26.87 ± 2.95	27.28 ± 3.11	0.900	0.369
Hypertension [n(%)]	55 (56.12%)	50 (60.98%)	0.433	0.511
Hyperlipidemia [n(%)]	44 (44.90%)	47 (57.32%)	2.755	0.097
Smoking history [n(%)]	28 (28.57%)	32 (39.02%)	2.195	0.138
Drinking history [n(%)]	31 (31.63%)	44 (53.66%)	8.911	0.003
Waist circumference (cm)	88.52 ± 5.24	89.45 ± 5.12	1.194	0.234
Course of disease (years)	5.02 ± 1.49	5.14 ± 1.56	0.549	0.583
Baseline HbA1c (%)	8.45 ± 1.18	8.92 ± 1.42	2.449	0.015
FBG (mmol/L)	10.24 ± 1.52	10.67 ± 1.61	1.820	0.070
TG (mmol/L)	2.78 ± 0.71	2.87 ± 0.69	0.874	0.383
HDL (mmol/L)	1.16 ± 0.21	1.06 ± 0.23	3.152	0.002

BMI, body mass index; HbA1c, glycated hemoglobin; FBG, fasting blood glucose; TG, triglyceride; HDL, high density lipoprotein.

#### 3.4.2 Management process indicators

**Figure 3:**
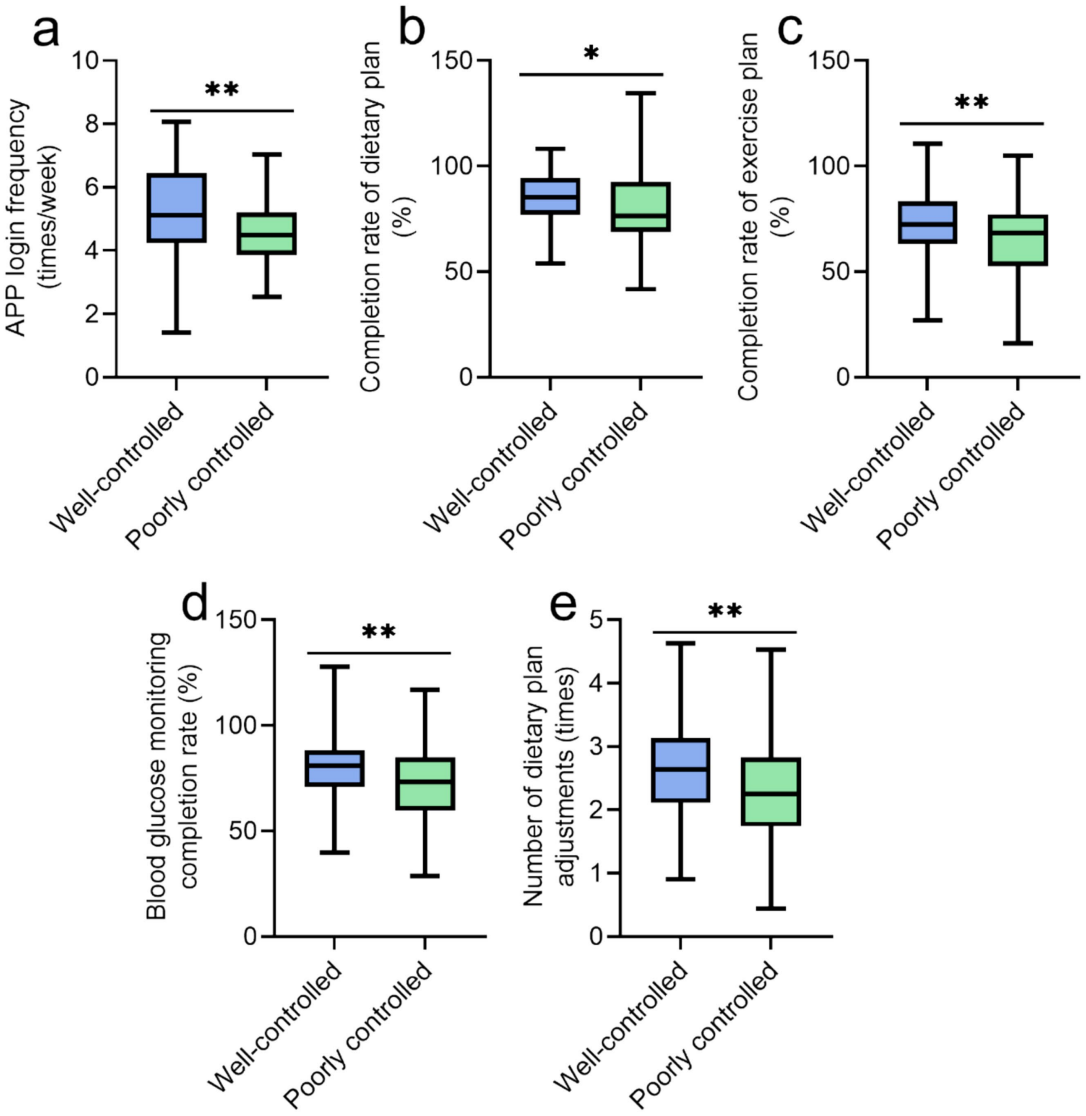
Comparison of management process indicators of the well-controlled group and the poorly controlled group under personalized management: **(a)** APP login frequency (times/week); **(b)** completion rate of dietary plan (%); **(c)** completion rate of exercise plan (%); **(d)** blood glucose monitoring completion rate (%); **(e)** number of dietary plan adjustments (times); *p < 0.05; **p < 0.01.

Figure 3 highlights significantly higher adherence in the well-controlled subgroup: more frequent APP logins (5.12 ± 1.46 vs. 4.54 ± 1.02 times/week, *p* = 0.002), better dietary (85.34 ± 11.27% vs. 80.15 ± 16.83%, *p* = 0.019) and exercise plan completion (72.45 ± 14.89% vs. 65.67 ± 18.24%, *p* = 0.007), higher blood glucose monitoring compliance (80.12 ± 13.45% vs. 72.34 ± 17.92%, *p* = 0.001) and number of dietary plan adjustments (2.65 ± 0.78 times vs. 2.32 ± 0.87 times, *p* = 0.008).

#### 3.4.3 Multivariate logistic regression analysis

Table 5 identifies alcohol consumption (OR = 3.576, *p* < 0.001), low HDL (OR = 0.102, *p* = 0.007), and reduced blood glucose monitoring adherence (OR = 0.958, *p* < 0.001) as independent risk factors for poor glycemic control. Exercise plan completion was protective (OR = 0.976, *p* = 0.037).

**Table 5:** Multivariate logistic regression analysis of the risk factors for poor blood glucose control under personalized management.

Indicators	Coefficient	Std error	Wald stat	*P*	OR	OR CI lower	OR CI upper
Drinking history [n(%)]	1.274	0.374	3.404	<0.001	3.576	1.717	7.449
Baseline HbA1c (%)	0.178	0.142	1.249	0.212	1.195	0.904	1.579
HDL (mmol/L)	−2.287	0.850	−2.691	0.007	0.102	0.019	0.537
APP login frequency (times/week)	−0.271	0.142	−1.916	0.055	0.762	0.578	1.006
Completion rate of dietary plan (%)	−0.024	0.012	−1.927	0.054	0.976	0.953	1.000
Completion rate of exercise plan (%)	−0.024	0.012	−2.083	0.037	0.976	0.954	0.999
Blood glucose monitoring completion rate (%)	−0.043	0.012	−3.471	<0.001	0.958	0.936	0.982
Number of dietary plan adjustments (times)	−0.401	0.218	−1.837	0.066	0.669	0.436	1.027

## 4 Discussion

This application study provides empirical evidence supporting the potential clinical utility of an AI and big data-driven personalized chronic disease management model for patients with T2DM. The findings demonstrate that integrating such a model into standard nursing-led care resulted in statistically significant improvements in glycemic control (FBG, 2hPG, HbA1c), enhanced self-care behaviors across all measured domains (SDSCA), and improved patient-reported quality of life (DSQL) over 6 months compared to conventional management, despite both groups being comparable at baseline. Furthermore, within the personalized management framework itself, factors associated with suboptimal glycemic control were identified, offering insights for future refinement.

### 4.1 Efficacy and mechanisms of personalized management

The superior glycemic outcomes observed in the personalized management group align with the growing body of research suggesting that tailored interventions outperform standardized approaches (22–24). The modest but statistically significant HbA1c reduction at 6 months, while below thresholds often considered clinically impactful in isolation (25), gains importance when viewed alongside the concurrent significant improvements in self-care behaviors and QoL. While the absolute differences observed in glycemic parameters (e.g., HbA1c reduction of 0.15%) may appear modest, they are consistent with the effect sizes reported in behavioral and lifestyle interventions delivered in real-world settings. According to (25), even a 0.1–0.2% reduction in HbA1c is associated with meaningful reductions in long-term complication risk, especially when sustained over time. Moreover, when these metabolic improvements are accompanied by significant enhancements in self-care behavior and quality of life—as observed in this study—they may reflect broader behavioral change and risk reduction. Therefore, although statistically modest, the results may carry clinical significance when considered in the broader context of chronic disease management. This pattern suggests the model may foster sustainable behavioral changes rather than merely achieving short-term metabolic shifts (23).

The largest improvement in the “special diet” domain of the SDSCA is particularly noteworthy. This likely stem directly from the model’s core functionality: dynamically generating individualized meal plans based on personal preferences (analyzed via app dietary logs), BMI, and activity levels. This contrasts sharply with conventional dietary advice, which often provides generic templates that fail to account for individual tastes and practical constraints, leading to poor long-term adherence (12). Our findings resonate with studies emphasizing the critical role of dietary personalization in T2DM management and the difficulty of sustaining generic recommendations (26, 27).

The significant improvements in exercise adherence, blood glucose monitoring, foot care, and medication taking, as captured by the SDSCA, further elucidate potential mechanisms for the glycemic benefits. The model’s ability to recommend contextually appropriate exercise types and intensities (considering age, comorbidities like hypertension, and habits) and to automate reminders likely reduced barriers to initiation and persistence. Similarly, the adaptive adjustment of glucose monitoring frequency based on recent fluctuations provided actionable feedback without unnecessary burden, potentially enhancing patient engagement and understanding of their glycemic patterns. These findings support the hypothesis that AI-driven models can overcome key limitations of episodic care by offering continuous, tailored support that adapts to individual needs and contexts in real-time, thereby bridging the gap between clinic visits (28, 29).

The superior quality of life outcomes across all DSQL dimensions (psychological, physiological, treatment, social) in the personalized group, particularly the marked improvement in psychological function, underscores a holistic benefit beyond metabolic control. This aligns with research linking personalized support, clear goals, and reduced regimen burden to improved psychological wellbeing in diabetes (30–32). This likely reflects reduced diabetes-related distress, a common barrier to effective self-management. The model’s features—providing specific, achievable plans, reducing uncertainty through adaptive feedback, and potentially increasing perceived control over the condition—may have alleviated anxiety and frustration associated with complex self-care regimens.

### 4.2 Factors influencing success within personalized management

The case–control analysis within the personalized management group revealed crucial insights into factors differentiating those achieving good versus poor glycemic control despite receiving the same advanced intervention. The identification of baseline low HDL-C and alcohol consumption as independent risk factors for poor control under personalized management reinforces established pathophysiological knowledge. Low HDL-C is a well-recognized component of diabetic dyslipidemia and insulin resistance, potentially rendering glycemic control more challenging irrespective of management intensity (33). Alcohol consumption can directly impair glycemic stability through effects on gluconeogenesis, insulin sensitivity, and hypoglycemia risk, while also potentially interfering with adherence to dietary and medication plans (33–35). These findings highlight that even sophisticated management models must contend with fundamental biological and behavioral factors.

More novel and specific to the digital, personalized approach were the findings related to engagement and adherence metrics. Lower adherence to the generated blood glucose monitoring plans emerged as a strong independent risk factor for poor control. This underscores the critical importance of acting upon the personalized recommendations; the model’s insights are only valuable if patients engage with the monitoring process that provides the necessary data for adaptation and feedback (36). Similarly, lower completion rates of the personalized exercise plans were associated with worse outcomes, emphasizing that tailored recommendations still require patient action. The protective effect of higher exercise plan adherence reinforces this point (37). These results highlight the essential role of adherence, not only to medication but also to behaviorally driven components like dietary and exercise plans, within AI-supported chronic disease management. Improving adherence in real-world practice may require multi-pronged strategies, such as personalized reminders, motivational messaging, adaptive goal-setting, and identifying early signs of disengagement through app usage analytics. AI models could be expanded to include adherence prediction modules, enabling proactive re-engagement interventions tailored to individual behavioral patterns. Interestingly, while APP login frequency and dietary plan adherence/completion rate showed significant univariate associations with better control, they did not retain significance as independent predictors in the multivariate model. This suggests that active engagement with the specific self-care actions (monitoring, exercising) prescribed by the model is more directly consequential for glycemic outcomes than general app usage or even dietary adherence within this specific framework, where dietary plans were highly personalized. The finding that patients requiring fewer dietary plan adjustments (potentially indicating initial plan suitability or stability) tended toward better control further hints at the interplay between personalization accuracy, patient engagement, and metabolic outcomes.

These process indicators offer a valuable new dimension for understanding intervention effectiveness in the digital health era. They move beyond simply measuring if an intervention is delivered to assessing how it is used and acted upon by the individual. This granular understanding of engagement is crucial for optimizing future iterations of AI-driven models, potentially allowing for early identification of individuals at risk of disengagement (e.g., low login frequency, declining adherence) and triggering proactive, tailored support strategies within the model itself (37, 38).

### 4.3 Limitations and implications

Several limitations warrant consideration. Firstly, the single-center, retrospective design limits generalizability and introduces potential selection bias. The requirement for smartphone literacy and internet access excludes digitally marginalized populations. The 6-month follow-up period is relatively short for assessing the long-term sustainability of benefits and prevention of chronic complications. Details of the AI model’s internal validation, feature importance for the XGBoost risk prediction, and the specific rules/reinforcement learning parameters within the plan generation submodel require fuller disclosure and external validation for reproducibility. The “black box” nature of complex AI models also poses challenges for clinical interpretability and trust.

Despite these limitations, this study provides promising evidence for the real-world application value of AI and big data in enhancing T2DM management. By demonstrating improvements in glycemic control, self-care, and QoL, alongside insights into key success factors within the model (engagement, adherence to specific actions, biological/behavioral baselines), it offers a foundation for future research. Larger, multi-center, prospective randomized controlled trials with longer follow-up are essential to confirm these findings and assess cost-effectiveness. Future work should focus on enhancing model interpretability, integrating predictive analytics for early intervention on adherence lapses identified through process indicators, and ensuring equitable access to such advanced digital health solutions. Integrating these AI tools as supportive complements within established, human-centered healthcare workflows, particularly empowering nursing-led management as demonstrated here, appears to be a promising pathway forward. Additionally, the cost of implementing and maintaining AI-driven tools and mobile health applications remains a relevant concern for real-world scalability. In this study, the mobile app and AI modules were developed in-house and deployed without cost to participants during the study period. However, future implementation may require resources for software maintenance, user support, and data security infrastructure. Although digital solutions may reduce long-term costs by improving outcomes and reducing complications, a formal cost-effectiveness analysis is necessary. Future studies should examine the economic feasibility of integrating such systems into routine care, particularly in resource-limited settings.

## 5 Conclusion

This study demonstrates the efficacy of an AI and big data-driven personalized management model in improving glycemic control, enhancing self-care activities, and elevating the quality of life for patients with T2DM. The personalized management group exhibited significantly better outcomes in fasting blood glucose, 2-h postprandial glucose, and HbA1c levels compared to the conventional management group. Furthermore, adherence to dietary and exercise plans, as well as blood glucose monitoring, was notably higher in the personalized group. These findings suggest that integrating advanced technologies into chronic disease management can lead to more effective and tailored interventions. Such an approach not only optimizes clinical outcomes but also supports patient empowerment and engagement, underscoring its potential for broader application in diabetes care.

## Data Availability

The raw data supporting the conclusions of this article will be made available by the authors, without undue reservation.
